# The Prognostic Significance of Protein Expression of CASZ1 in Clear Cell Renal Cell Carcinoma

**DOI:** 10.1155/2019/1342161

**Published:** 2019-08-06

**Authors:** Bohyun Kim, Minsun Jung, Kyung Chul Moon

**Affiliations:** Department of Pathology, Seoul National University College of Medicine, Republic of Korea

## Abstract

**Backgrounds:**

Clear cell renal cell carcinoma (ccRCC) is the most common histologic subtype of renal cell carcinoma (RCC) and shows a relatively poor prognosis among RCCs. Castor zinc finger 1 (CASZ1) is a transcription factor, prominently known for its tumor suppression role in neuroblastoma and other cancers. However, there has been no research about the prognostic significance of CASZ1 in ccRCC. In this study, we investigated CASZ1 expression in ccRCC and analyzed its prognostic implications.

**Methods:**

A total of 896 ccRCC patients, who underwent surgical resection from 1995 to 2008, were included. We prepared tissue microarray blocks, evaluated CASZ1 nuclear expression by immunohistochemistry, and classified the cases into low or high expression categories.

**Results:**

A low expression of CASZ1 was observed in 320 cases (35.7%) and was significantly associated with large tumor size, high World Health Organization/International Society of Urological Pathology (WHO/ISUP) grade, and high T category and M category. In survival analysis, a low expression of CASZ1 was significantly correlated with unfavorable progression-free survival (PFS) (*p* < 0.001), overall survival (OS) (*p* < 0.001), and cancer-specific survival (CSS) (*p* < 0.001) and was an independent prognostic factor for PFS and CSS in multivariate analysis adjusted for tumor size, WHO/ISUP grade, T category, N category, and M category.

**Conclusions:**

Our study is the first to show the prognostic significance of CASZ1 expression in ccRCC. Our results revealed that low expression of CASZ1 is associated with poor prognosis and may serve as a new prognostic indicator.

## 1. Introduction

Kidney cancer is the 15th most common cancer and the 17th most common cause of cancer-related death worldwide [1]. Renal cell carcinoma (RCC) is the most common malignant kidney tumor [2], and its incidence is increasing [3]. RCC is a heterogenous group of carcinomas that includes a clear cell subtype, a papillary subtype, and a chromophobe subtype [4]. Each subtype differs in histological characteristics, aggressiveness, and prognosis [5]. The most common histological subtype is the clear cell type, which makes up 80% of all RCCs [6]. For clear cell RCC (ccRCC), surgical excision is the primary treatment option, and in cases of surgically unresectable tumors or in cases of recurrence, pazopanib or sunitinib is used as first-line therapy [7].

Castor zinc finger 1 (CASZ1) is a transcription factor that has been reported to play an important role in neural and cardiac development [8, 9]. Some studies have suggested that CASZ1 induces vascular assembly and morphogenesis [10, 11]. Recent studies reported that CASZ1 regulates T helper cell plasticity and has important implications for autoimmune inflammation [12]. Some studies showed that CASZ1 has a role in tumor progression. CASZ1 regulates tumor growth and the process of development and thus can be a candidate for tumor suppression in neuroblastomas [13, 14]. Additionally, loss of CASZ1 is associated with poor prognosis in neuroblastomas [15]. CASZ1 was found to be significantly hypermethylated in esophageal squamous cell carcinoma [16]. A CASZ1-MASP2 fusion transcript was identified in colorectal cancer with 3′ overexpression of MASP2 [17]. CASZ1 downregulation was correlated with aggressiveness and poor outcome in hepatocellular carcinoma (HCC) [18]. On the other hand, CASZ1 promoted the epithelial-mesenchymal transition and cancer metastasis in epithelial ovary cancer [19]. CASZ1 possibly plays different roles in various cancers. It has been reported that the expression of CASZ1 is downregulated in ccRCC tissue [13], but no study so far has investigated the relationship between CASZ1 expression and prognosis in ccRCC. Thus, in this study, we investigated CASZ1 expression in ccRCC and analyzed its prognostic value.

## 2. Materials and Methods

### 2.1. Patients and Tissue Samples

In total, ccRCC tissues from 896 patients who underwent surgical resection at Seoul National University Hospital (SNUH) from 1995 to 2008 were included in this study. We searched the computerized database of the Department of Pathology, SNUH, and we retrospectively collected clinical and pathologic information from medical records and pathologic reports. We reviewed hematoxylin and eosin- (H&E-) stained slides to confirm the diagnosis and to identify various pathologic parameters.

A tissue microarray (TMA) block was prepared from formalin-fixed paraffin-embedded tissue blocks (SuperBioChips Laboratories, Seoul, Republic of Korea). For each case, two tumor cores (2 mm in diameter) were collected. Each core was derived from different tumor areas with representative clear cell histology.

This study was approved by the Institutional Review Board (IRB) of SNUH (IRB No H-1903-149-1022) and was performed in accordance with the principles of the Declaration of Helsinki.

### 2.2. Immunohistochemistry (IHC)

For immunohistochemical analyses, the TMA blocks were cut at 4 *μ*m thickness. A rabbit anti-CASZ1 polyclonal antibody (Novus Biologicals, Centennial, CO, USA) was used at a dilution of 1 : 100. IHC was performed using the Ventana Benchmark XT automated staining system (Ventana Medical Systems, Tucson, AZ, USA) according to the manufacturer's instructions.

### 2.3. Immunohistochemical Scoring

**Figure 1:**
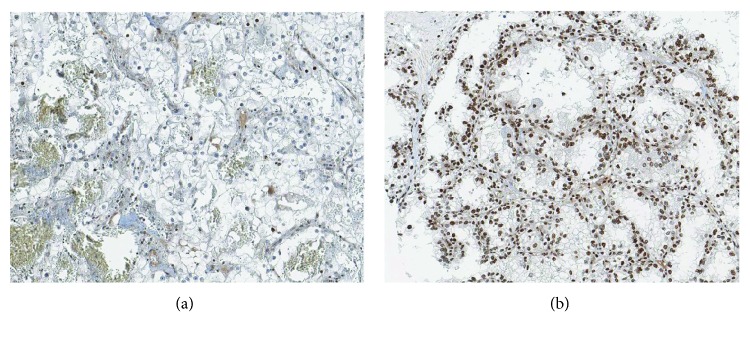
Immunohistochemical expression of CASZ1 in clear cell renal cell carcinoma. Representative images of low (a) and high (b) expressions (×200).

CASZ1 immunohistochemical staining was mainly localized in the nucleus in positive cases. CASZ1 protein expression was evaluated by the percentage of positively stained cells. The percentage of stained cells is the ratio of the number of tumor cells with positive CASZ1 staining to the total number of tumor cells in the TMA tumor core area. The percentage of stained cells was scored 0 to 5+ (0: no tumor cell staining, 1+: <1%, 2+: 1%-10%, 3+: 11%-33%, 4+: 34%-66%, and 5+: 67%-100%). For each case, both cores were evaluated, and the mean value was used for statistical analysis. In receiver operating curve analysis, we discerned the optimal cut-off value with the highest Youden index [20]. A score of 2.5 was used as a cut-off value to classify all cases as either high CASZ1 expression or low CASZ1 expression. Representative images of high and low CASZ1 expressions are shown in Figure 1. One pathologist (B.K.) evaluated CASZ1 staining at two different time points, without awareness of the previous results at the second evaluation. Any cases with discrepant results were reviewed together with another pathologist (K.C.M.) for final scoring.

### 2.4. Statistical Analysis

The follow-up period was the time between the surgery and the last follow-up. The progression-free survival (PFS) period was defined as the time period between the time of surgery and the time of recurrence at the operation site, lymph node metastasis, distant metastasis, or death by clear cell renal cell carcinoma. The overall survival (OS) period was defined as the time period between the time of surgery and the time of death, or it was censored at the time of the last follow-up. The cancer-specific survival (CSS) period was defined as the time period between the time of surgery and the time of cancer-related death, or it was censored at the time of the last follow-up.

The association between CASZ1 expression and the patient's clinicopathologic characteristics was evaluated by the chi-squared test. The associations between CASZ1 expression and PFS, CSS, and OS were evaluated by the Kaplan-Meier method with the log-rank test. The significance of covariates was evaluated by the univariate Cox proportional hazards model. Multivariate analysis was performed with covariates which showed statistical significance on univariate analysis. Statistical analyses were performed using SPSS software (version 23; IBM, Armonk, NY, USA). Two-sided *p* values of <0.05 were considered to be statistically significant.

## 3. Results

### 3.1. Clinicopathologic Characteristics of Patients

Overall, 896 patients were included in this study, including 671 men and 225 women. The age of the patients ranged from 20 to 84 years, with the mean age of 56 years. The diameter of primary tumors ranged from 5 to 220 mm, with the mean diameter of 47 mm. Lymph node metastasis was found in 16 cases (1.8%), and distant metastasis was found in 68 cases (7.6%). According to the 8th edition of the TNM staging system of the AJCC [21], 607 patients were in stage I, 77 patients in stage II, 139 patients in stage III, and 73 patients in stage IV. According to the WHO/ISUP grading system [22], 53 cases were classified as grade 1, 406 cases as grade 2, 360 cases as grade 3, and 77 cases as grade 4. There was no case that underwent neoadjuvant therapy. Sixty-eight cases underwent adjuvant therapy, which included 57 cases with distant metastasis at the time of surgery and some cases with T category 4 or with lymph node metastasis. A high expression of CASZ1 was observed in 83.6% (749/896); a low expression of CASZ1 was observed in 16.4% (147/896).

### 3.2. Association of CASZ1 Expression with Clinicopathologic Characteristics

Table 1 summarizes the clinical and pathologic characteristics of the 896 cases. The low expression of CASZ1 was significantly correlated with old age (>55 years) (*p* = 0.037), large tumor size (*p* = 0.004), high WHO/ISUP grade (*p* < 0.001), and high T category (*p* < 0.001) and M category (*p* = 0.008) but not correlated with gender or N category.

### 3.3. Association of CASZ1 Expression with Prognosis

**Figure 2:**
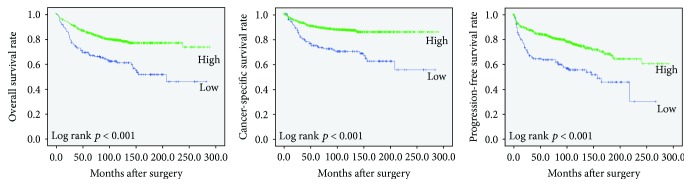
Kaplan-Meier curves for impact of the CASZ1 expression on overall survival, cancer-specific survival, and progression-free survival.

The follow-up period ranged from 1 to 288 months, and the median follow-up period was 94 months. During the follow-up period, disease progression was found in 117 cases (13.1%) and cancer-related death occurred in 53 cases (5.9%). Kaplan-Meier analysis showed that the low expression of CASZ1 was associated with unfavorable OS, CSS, and PFS (*p* < 0.001, *p* < 0.001, and *p* < 0.001, respectively) (Figure 2).

### 3.4. Univariate and Multivariate Analyses of Clinicopathologic Parameters and Expression of CASZ1

Cox proportional hazards analysis was performed to analyze the risk factors associated with the survival of ccRCC patients. The results are summarized in Tables 2 and 3. In univariate analysis, the low expression of CASZ1 was a significant risk factor for unfavorable OS (*p* < 0.001), CSS (*p* < 0.001), and PFS (*p* < 0.001). Additionally, a high WHO/ISUP grade and high T category, N category, and M category were significant risk factors for unfavorable OS, CCS, and PFS. Multivariate analysis was performed with risk factors that were statistically significant on univariate analysis. The CASZ1 expression and high WHO/ISUP grade, T category, N category, and M category were independent prognostic factors for OS, CSS, and PFS.

## 4. Discussion

In this study, we analyzed the prognostic value of CASZ1 protein expression in ccRCC. We performed immunohistochemical staining of CASZ1 in 896 ccRCC cases and demonstrated that a low expression of CASZ1 was associated significantly with advanced clinicopathologic parameters of ccRCC such as large tumor size, high WHO/ISUP grade, high T category and M category, and high TNM staging. Collectively, our study results show that the low expression of CASZ1 is significantly associated with shorter PFS, OS, and CSS in patients with ccRCC. In multivariate analysis adjusted for nuclear grade and overall stage, the low expression of CASZ1 is an independent prognostic parameter for shorter PFS and CSS of patients with ccRCC.

Great progress has been reported in the research about tumorigenesis, management, and treatment of ccRCC, but little has been discovered about its clinical biomarkers except for the pathologic stage and microscopic necrosis. CASZ1 is a transcription factor, and few studies have revealed the expression of CASZ1 in tumors. In neuroblastoma and HCC, CASZ1 expression was lower in aggressive stage tumors or in cases with poor prognosis [15, 18]. In ovary epithelial cancer, on the other hand, CASZ1 expression was higher in metastatic tumors [19]. These results indicate that CASZ1 has different tumor-specific roles in different tumor types. In ccRCC, based on our study, we suggest that a decreased CASZ1 expression seems to be correlated with tumor progression. To the best of our knowledge, our study is the first to demonstrate the association of CASZ1 protein expression with ccRCC clinicopathologic correlation and prognosis.

Liu et al. suggested that CASZ1 activated pRb in the G1 cell cycle, thus inhibiting cell cycle progression, and reported that in the gene set enrichment assay, CASZ1 repressed MYC target genes in neuroblastoma [23, 24].

Wang et al. suggested that CASZ1 inhibits the MAPK/ERK signaling pathway by downregulating RAF1 in HCC. These authors performed a Cignal Finder Cancer 10-Pathway Reporter Array experiment and showed that the MAPK/ERK pathway was the most affected. Immunohistochemistry expression of phosphorylated-ERK (p-ERK), MMP2, MMP9, and cyclin D1, which are regulated by the MAPK/ERK signaling pathway, was increased in CASZ1-silenced cells. Furthermore, these authors demonstrated that CASZ1 inhibits RAF1 protein expression, which is an important element of the MAPK signaling pathway [18]. Furthermore, overexpression of p-ERK is associated with adverse prognosis in RCC [25, 26]. These studies, together with our findings, indicate that CASZ1 can be considered to inhibit RCC progression by inhibiting the MAPK/ERK signaling pathway.

Charpentier et al. suggested that CASZ1 is required for vascular patterning and lumen formation [10, 11, 27, 28]. These authors demonstrated that CASZ1 regulates epidermal growth factor-like domain 7 (Egfl7) and miR-126 to control angiogenesis and vascular remodeling in human cells. CASZ1 positively induces Egfl7 and miR-126 expression in human cells, and so, its depletion leads to altered morphology and cell adhesion in human vascular endothelial cells [10]. Numerous studies have investigated microRNAs (miRNA), which are noncoding RNAs, and their role in the regulation of gene expression [29, 30]. Many of these studies have suggested that miR-126 inhibits cancer cell proliferation and invasion and is correlated with favorable prognosis in various human cancers [31–35]. The low expression of miR-126 is associated with shorter CSS and OS in RCC [36], with metastasis in ccRCC [37, 38], and with therapeutic resistance and cell motility in RCC [39]. The high expression of miR-126 is associated with significantly longer disease-free survival and OS [40] and with low WHO/ISUP grade [41].

## 5. Conclusion

In summary, this study suggests that CASZ1 could be a potential biomarker for predicting the aggressiveness of ccRCC. Further functional studies are needed to validate this role of CASZ1 and to identify the mechanism of CASZ1-mediated tumor progression.

## Figures and Tables

**Table 1:** Clinicopathologic characteristics of patients and association with CASZ1 expression.

	CASZ1 expression		
	Low *N* (%)	High *N* (%)	*p* value
Age (years)			
≤55	58 (39.5%)	366 (48.9%)	0.037
>55	89 (60.5%)	383 (51.1%)	
Gender			
Male	111 (75.5%)	560 (74.8%)	0.849
Female	36 (24.5%)	189 (25.2%)	
Tumor size (cm)			
≤7	100 (68.0%)	592 (79.0%)	0.004
>7	47 (32.0%)	157 (21.0%)	
WHO/ISUP grade			
Grades 1-2	54 (36.7%)	405 (54.1%)	<0.001
Grades 3-4	93 (63.3%)	344 (45.9%)	
T category			
T1-T2	101 (68.7%)	619 (82.6%)	<0.001
T3-T4	46 (31.3%)	130 (17.4%)	
N category			
N0/Nx	143 (97.3%)	737 (98.4%)	0.349
N1	4 (2.7%)	12 (1.6%)	
M category			
M0	182 (87.1%)	700 (93.5%)	0.008
M1	19 (12.9%)	49 (6.5%)	

**Table 2:** Univariate analysis of overall, cancer-specific, and progression-free survival.

	OS		CSS		PFS	
	HR (95% CI)	*p* value	HR (95% CI)	*p* value	HR (95% CI)	*p* value
CASZ1						
Low vs. high	0.441 (0.328-0.593)	<0.001	0.363 (0.254-0.518)	<0.001	0.456 (0.342-0.608)	<0.001
Age (years)						
≤55 vs. >55	3.242 (2.383-4.410)	<0.001	2.290 (1.596-3.285)	<0.001	1.852 (1.420-2.415)	<0.001
Gender						
Male vs. female	1.160 (0.845-1.594)	0.359	1.149 (0.771-1.714)	0.494	1.0800 (0.800-1.457)	0.617
WHO/ISUP grade						
1, 2 vs. 3, 4	2.650 (1.994-3.520)	<0.001	6.093 (3.892-9.538)	<0.001	3.034 (2.295-4.012)	<0.001
T category						
T1, T2 vs. T3, T4	3.413 (2.597-4.485)	<0.001	6.012 (4.298-8.4.9)	<0.001	4.822 (3.715-6.259)	<0.001
N category						
N0/Nx vs. N1	8.100 (4.596-14.278)	<0.001	10.984 (6.026-20.018)	<0.001	6.655 (3.710-11.938)	<0.001
M category						
M0 vs. M1	11.088 (8.098-15.182)	<0.001	19.696 (13.821-28.067)	<0.001	15.998 (11.726-21.826)	<0.001

OS: overall survival; CSS: cancer-specific survival; PFS: progression-free survival; HR: hazard ratio; CI: confidence interval.

**Table 3:** Multivariate analysis of overall, cancer-specific, and progression-free survival.

	OS		CSS		PFS	
	HR (95% CI)	*p* value	HR (95% CI)	*p* value	HR (95% CI)	*p* value
CASZ1						
Low vs. high	0.589 (0.437-0.794)	0.001	0.526 (0.367-0.754)	<0.001	0.558 (0.417-0.747)	<0.001
WHO/ISUP grade						
1, 2 vs. 3, 4	1.775 (1.317-2.391)	<0.001	3.387 (2.129-5.389)	<0.001	1.866 (1.386-2.513)	<0.001
T category						
T1, T2 vs. T3, T4	1.855 (1.378-2.498)	<0.001	2.538 (1.768-3.645)	<0.001	2.431 (1.813-3.259)	<0.001
N category						
N0/Nx vs. N1	5.417 (3.037-9.662)	<0.001	6.190 (3.346-11.451)	<0.001	3.918 (2.157-7.116)	<0.001
M category						
M0 vs. M1	6.497 (4.636-9.105)	<0.001	9.385 (6.429-13.702)	<0.001	7.677 (5.469-10.778)	<0.001

OS: overall survival; CSS: cancer-specific survival; PFS: progression-free survival; HR: hazard ratio; CI: confidence interval.

## Data Availability

The data used to support the findings of this study are available from the corresponding author upon request.
